# T Cells in Fish

**DOI:** 10.3390/biology4040640

**Published:** 2015-09-25

**Authors:** Teruyuki Nakanishi, Yasuhiro Shibasaki, Yuta Matsuura

**Affiliations:** Department of Veterinary Medicine, Nihon University, Fujisawa, Kanagawa 252-0880, Japan; E-Mails: yasuhiroshibasaki@gmail.com (Y.S.); bryu13505@g.nihon-u.ac.jp (Y.M.)

**Keywords:** T cells, CD4, CD8, Thymus, Cytotoxicity

## Abstract

Cartilaginous and bony fish are the most primitive vertebrates with a thymus, and possess T cells equivalent to those in mammals. There are a number of studies in fish demonstrating that the thymus is the essential organ for development of T lymphocytes from early thymocyte progenitors to functionally competent T cells. A high number of T cells in the intestine and gills has been reported in several fish species. Involvement of CD4^+^ and CD8α^+^ T cells in allograft rejection and graft-*versus*-host reaction (GVHR) has been demonstrated using monoclonal antibodies. Conservation of CD4^+^ helper T cell functions among teleost fishes has been suggested in a number studies employing mixed leukocyte culture (MLC) and hapten/carrier effect. Alloantigen- and virus-specific cytotoxicity has also been demonstrated in ginbuna and rainbow trout. Furthermore, the important role of cell-mediated immunity rather than humoral immunity has been reported in the protection against intracellular bacterial infection. Recently, the direct antibacterial activity of CD8α^+^, CD4^+^ T-cells and sIgM^+^ cells in fish has been reported. In this review, we summarize the recent progress in T cell research focusing on the tissue distribution and function of fish T cells.

## 1. Introduction

T cells play important roles in the adaptive immune system. All T cells possess a T cell receptor (TCR) by which they recognize peptide presented by MHC, along with CD3 and co-stimulatory (e.g., CD28) and co-inhibitory (e.g., CTLA-4) surface molecules. Mammalian TCR comes mainly in two forms: A heterodimer of TCRα and TCRβ chains is found on the surface of conventional circulating αβ-T cells, and a heterodimer of TCRγ and TCRδ chains is found on the “more primitive” mucosa-associated γδ-T cells. αβ-T cells are the more abundant T cell type found in lymphoid organs and blood in mammals. Recently, a new additional TCR chain called TCRμ was discovered in marsupials [1] and monotremes [2]. TCRμ does not have a known homolog in placental mammals and nonmammals but has features analogous to a TCRδ isoform in sharks.

T cells are categorized into two general populations according to their function, cytotoxic T cells (CTLs) and helper T (Th) cells. CTLs express CD8 molecules involved in the interaction with MHC class I, while helper T cells express CD4 that interacts with MHC class II. Recently, in humans and mice, helper T cells are further divided into several populations, Th1, Th2, Th17 and Tregs which play different roles in immune responses.

T cell associated genes and their encoded proteins with T cell activity, e.g., surface markers, cytokines and transcriptional factors, have been well documented (reviewed by [3,4]). In the present review, we summarize the recent progress in T cell research focusing on the tissue distribution and function of fish T cells.

## 2. Identification of T Cell Populations in Fish

The pesence of CTLs and Th cells in fish has been suggested in a number of functional studies (reviewed by [4]), and recently CTLs and Th have been identified as CD8^+^ and CD4^+^ cells, respectively using monoclonal antibodies (mAbs). In earlier studies, the presence or function of T cells was inferred as or associated with surface Ig (sIg) negative cells using mAbs against IgM which are available for many fish species. MAbs against fish T cells have been produced in only a few species, e.g., carp (WCT, WCL9, WCL38) and seabass (DLT15) (see review by [5]). Antibodies against T cell specific surface antigens that are well conserved throughout vertebrates have been also used to identify fish T cells. CD3ε and ZAP70 are well conserved and antibodies against the intra-cellular domain of human CD3ε and ZAP70 have been used to identify T cells in fixed cells and tissues of several fish species, e.g., CD3ε for Atlantic salmon [6], ZAP70 for carp [7] and zebrafish [8]. T cells have been also histologically identified by ISH detecting mRNAs of *tcr*, *cd4*, *cd8*, *etc.* Transgenic animals harboring the GFP gene downstream of the *lck* promotor have been produced and used to identify and isolate T cells in zebrafish [9]. Using antibodies or antibodies in combination with mRNA expression analysis, the tissue distribution and function of fish T cells have been investigated in several fish species as described below.

However, identification and isolation of CD4^+^ and CD8^+^ T cells have not been possible until the work by our group who succeeded in producing mAbs against CD4 and CD8α in ginbuna crucian carp [10,11]. Recently, the techniques for mAb production have been applied to rainbow trout where mAbs against CD4-1, CD4-2, CD8α and CD8β are available (Takizawa *et al.* [12] for CD8α, personal communication for others). With the aid of mAbs against T cell subsets, CD8^+^ T cells have been identified as CTLs and the helper function of CD4^+^ T cells has been demonstrated [10,11]. It is noteworthy that CD4 and CD8 molecules are expressed not only on T cells but also other cell types, e.g., CD4-1 in melano-macrophages in channel catfish [13] as in the case of CD4 and CD8 expression by human and mouse thymic dendritic cells [14]. Therefore, multiple markers should be used for the true identification of T cells.

Regulatory T cells (Tregs) are defined as CD4^+^CD25^+^ T cells expressing the transcription factor forkhead box P3 (Foxp3) in charge of maintaining immunological unresponsiveness to self-antigens and in suppressing excessive immune responses deleterious to the host. Tregs have diverse roles in numerous diseases, including autoimmunity, allergy and cancer. Treg-like cells with the phenotype CD4-2^+^, CD25-like^+^, Foxp3-like^+^ showing a suppressive effect on mixed leukocyte culture (MLC) and nonspecific cytotoxic cell (NCC) activity *in vitro* have been reported in pufferfish [15]. However, not *cd25* (*il2rα*) but *il15rα* is present in bony fish [16] and function of fish Foxp3 is a matter of discussion. Therefore, the presence of true Treg is in question and further studies are required.

## 3. Development of T Cells and Thymus

The thymus is a specialized primary lymphoid organ of the immune system where T cells develop and mature, and is composed of two lobes in most mammals but more than two in sharks, amphibians, birds and in some teleost fishes [17,18]. Histologically, each lobe in most of the mammalian thymus is composed of numerous lobules which are divided into a peripheral cortex and a central medulla.

Cartilaginous and bony fish are the most primitive vertebrates with a histologically identifiable thymus. The thymus in most teleosts is located near the gill cavity and present even in adult fish, although the volume diminishes with age or sexual maturation. In general, teleost thymus tends to lack a clear corticomedullary regionalization (reviewed by [19,20]. Thymus contains distinct cortical and medullary regions in ciclids and cyprinids [21,22] but this distinction was not made in other species [23]. In zebrafish, a morphological distinction between cortex and medulla was not noted by Willett *et al.* [24]. However, it was found subsequently that rag1 transcripts are located only in peripheral regions of the zebrafish thymus, presumably corresponding to the cortex, whereas TCRα transcripts are distributed throughout the thymus [25]. Very recently, thymus-like lympho-epithelial structures, termed thymoids, have been reported in the gill filaments and the neighbouring secondary lamellae of lamprey larvae, although the presence of distinct cortex and medulla structure has not been studied [26].

During the development of many teleost species, the thymus is the first lymphoid organ to develop and the first to become lymphoid. This is followed by the kidney, with the spleen developing later and remaining predominantly erythroid throughout life (reviewed by [27]). However, the appearance of thymic rudiment and lymphocytes varies between species due to differences in classification of embryonic stages and rearing temperatures, although there is a general pattern to the sequential development of the lymphoid organs described above. For instance, in rainbow trout the thymus is present as a rudiment at five days pre-hatch at 14 °C [28]. In contrast, in an ovoviviparous marine teleost *Sebastiscus marmoratus,* the rudiment of the thymus was first visible 10–12 days post-hatch (seven days post-birth) at 20 °C, while the kidney and the spleen were differentiated at the time of birth and contained small numbers of haemopoietic cells [29]. Similar findings with late appearance of the thymus have been reported in other marine teleosts [30], although lymphocytes first appear in the thymus (Table 1).

**Table 1:** Development of thymus and T cells in fish.

Species, temperature in parenthesis	Formation of thymic rudiment	Appearance of small lymphocytes	Expression of T cell relevant genes in thymus	Differentiation of Cortex/Medulla	References
Rainbow trout(14°C) (8–15°C)	5 days pre-hatch (stage 28 *^1^)	3 dph (stage 32 *^1^)	*cd8α*: 1wpf *tcrβ*: 2 wpf		[28] [31]
Carp (22–25°C) Hatching occurs 2–3.5 days after fertilization	2 dph (stage 27–31 *^2^) 3 dpf	8 dph (stage 35–36 *^2^) 4 dpf (WCL9+ve cells)	*rag1*: 4 dpf	4 wpf 1 wpf (*rag1*/WCL9)	[32] [22] [33]
Zebrafish (27–28.5 °C) Hatching occurs 3 days after fertilization	54–60 hpf	7 dpf	*ikaros*, *gata3*, *rag1*, *lck*: 72 hpf 3.5dpf *tcrα* & *rag1*: 4 dpf	6 wph (*rag1* expression at peripheral thymus)	[34] [35] [25]
Sea bass (15–16 °C) Hatching occurs 2 days after fertilization	27 dph	30 dph (DLT15+ve cells)	*tcrβ*: 25 dph *cd4, cd8α*: 51 dph	75 dph (*cd4* expression)	[36] [38,39]
Rock fish (20 °C) Birth occurs 4 days after hatching	10–12 dph	21 dph	ND	40 dph	[29]

hpf: hours post-fertilization; dpf: days post-fertilization; dph: days post-hatch; wpf: weeks post-fertilization; wph: weeks post-hatch. *^1^: Developmental stages for rainbow trout were designated according to Vernier (1969). *^2^: Developmental stages for carp were designated according to Balinsky (1948).

There are numerous studies with regard to the development of T cells and the thymus in zebrafish. The thymic rudiment is formed by 60 h post-fertilization (hpf) followed by the identification of lymphoblasts by electron microscopy at 65 hpf [34]. Expression of *ikaros* which in mammals is expressed in lymphoid progenitors, and the recombination activating genes, *rag1* which is required for the differentiation of B and T lymphocytes, is detected in zebrafish thymus at 3.5 dpf [35]. TCRα expression was first detected by ISH and RT-PCR at four days post-fertilization (dpf) in the thymus. At six weeks, TCRα was expressed throughout the thymus, whereas rag1 expression was localized to the peripheral regions [25]. No distinction into cortex and medulla is observed until three weeks post-fertilization (wpf). Immunocompetence in zebrafish, as measured by humoral response to T-dependent and -independent antigens, is not reached until 4–6 wpf [37].

Similar to zebrafish, in carp the appearance of thymic primordium occurs at 3 dpf along with *rag1* expression in embryo heads. Expression of *rag1* and WCL9 mAb (cortical thymocytes) positive cells were found at 4 dpf in the thymus, and both rag-1^+^/WCL9^+^ and rag-1^−^/WCL9^−^ areas were distinguished from 1 wpf, suggesting early cortex/medulla differentiation [33]. From 1 wpf, *rag1*/*rag2* was expressed in kidney but not in spleen, while WCI12 (IgM^+^ B cells)^+^ cells appeared one week later in both organs, suggesting IgM^+^ B cell recombination in kidney but not in spleen. Interestingly, *rag1*/*rag2* expression was detected in thymus of carp over over-year-old, but in kidney only at low levels, indicating life-long new formation of putative T cells [33].

Picchietti *et al.* [38,39] investigated the gene expression of *tcrβ*, *cd4-1* (only one form of CD4 presents in this species lacking CD4-2) and *cd8α* in sea bass during ontogeneic development. TCRβ mRNA was detected in the larvae on day 25 post-hatch (= 27 dpf) and CD8α transcripts 26 days later (= 53 dpf). Using ISH at day 51 ph (= 53 dpf), CD8α, CD4-1 and TCRβ mRNAs were localized in thymocytes of the outer and lateral zones of the thymus. From day 75 ph (= 77 dpf) onwards the signals were mainly detected in the outer region, drawing a cortex-medulla demarcation. In one-year-old fish, CD8α^+^ and TCRβ^+^ thymocytes almost completely filled the cortex and extended in large cords into the medulla.

## 4. Distribution of T Cells in Tissues

Mature T cells are distributed throughout the body particularly in lymphoid tissues such as the thymus, kidney in teleost and spleen. Recently, abundant presence of T cells was identified in mucosal tissues such as the intestine, gill and skin.

### 4.1. Lymphoid Tissues (Thymus, Kidney, Spleen)

It is well documented in mammals that the thymus is the essential organ for development of T lymphocytes from early thymocyte progenitors to functionally competent T cells [40,41]. Earlier studies with fish investigated the distribution of T cells by *in situ* hybridization (ISH) using T cell-specific markers. Araki *et al.* [42] reported that CD3-expressing cells in fugu were restricted to the lymphoid outer zone and epithelioid inner zone of the thymus, while those cells were distributed randomly in the head kidney, trunk kidney, and spleen. Romano *et al.* [43] revealed the distribution of T cells in the sea bass thymus using mAb DLT15 (pan-T-cell marker) in combination with ISH. Namely, DLT15^+^ and TCRβ^+^ cell populations were concentrated in the cortex and TCRβ^+^ cells were reactive at the cortical-medullary border. Accordingly, these data suggest that outer and inner thymic zones in fish correspond to cortex and medulla in mammalian thymus, respectively.

In adult sea bass, Picchietti *et al.* [39] observed that CD4^+^ and CD8α^+^ double positive cells (DP) thymocytes filled the thymic cortex and expression patterns of CD4 and CD8α largely overlapped in the cortex, while CD4^+^ or CD8α^+^ single positive cells (SP) were differently distributed in the medulla. These observations reflect T lymphocyte differentiation pathways similar to those in mammals.

Toda *et al.* [11] reported the presence but not distribution of T cell subsets in ginbuna thymus using monoclonal antibodies against CD4 (mAb 6D1) and CD8α (mAb 2C3). They demonstrated that CD4/CD8 DP were present only in the thymus and that the percentages of DN, DP, CD4 SP and CD8 SP were approximately 37%, 16%, 29% and 19% of total thymocytes, respectively. In mice it has been reported that DN, DP, CD4 SP and CD8 SP constitute 5%, 80%–85%, 10% and 5% of the total thymocytes, respectively [44]. The percentage of DP in the ginbuna thymus seems to be very low compared with that in mice. Somamoto *et al.* [45] reported that mAb 6D1 recognizes CD4-1 (immunogen) but not CD4-2 (CD4 rel, fish-specific CD4) of which structure is considerably different. Similarly, there are at least two isoforms of CD8α (CD8α-1 and CD8α-2) in ginbuna. Accordingly, the lower percentage of DP cells in ginbuna thymus may be due to only counting CD4-1/ CD8α-1 double positive cells as DP cells, excluding any CD4-2 or CD8α-2 positive cells that may be present.

### 4.2. Mucosal Tissues (Intestine, Gill)

Early studies suggested the presence of T cells in mucosal tissues [46] using a mAb against carp intestinal T cells (WCL38). WCL38^+^ cells were abundant in the intestinal epithelium and less numerous in the lamina propria and also reacted with lymphoid cells in gills and skin.

To be interested, the presence of gradient in the number of lymphocytes has been reported even in developing sea bass [47]. Namely, mAb DLT15^+^ cells increase in concentration towards the anus. This phenomenon coincides well with the importance of the posterior (second) gut for antigen uptake and transport in other fish species (see review by [48]). Romano *et al.* [43] also confirmed that the density of DLT15^+^ T cells increased from the anterior to posterior intestine, whereas TCRβ^+^ lymphocytes were more numerous in the middle intestine compared with other segments. The concentration of TCRβ^+^ cells in the sea bass midgut also strongly suggests a special role for this intestinal segment in antigen-specific cellular immunity. The authors suspected that the large population of TCRβ (-)/DLT15^+^ T cells in the posterior gut may be TcRγδ T cells.

Picchietti *et al.* [49] reported the presence of *cd8α* expression in the posterior segment of the sea bass intestine. They also found that TCRβ and CD8α transcript levels exceeded those of CD4-1 in the whole intestine, and confirmed by ISH that mucosal CD8α^+^ cells were especially numerous in the epithelium and in aggregates in the lamina propria. Furthermore, high non-specific cytotoxic activity against xenogeneic and allogeneic cells was found in lymphocytes from the intestinal mucosa.

Using rabbit sera recognizing a peptide sequence of the CD3ε chain, Koppang *et al.* [6] confirmed high numbers of CD3ε^+^ or T cells in the epithelium of intestinal tract as well as the thymus and gill of Atlantic salmon. Bernard *et al.* [50] suggested that rainbow trout intraepithelial lymphocytes (IELs) contain primarily T cells with unique TCR repertoire being highly diverse and polyclonal in naive adult individuals, in sharp contrast with the restricted diversity of IEL oligoclonal repertoires described in birds and mammals (see review by Dr. Salinus in this issue for details). Interestingly, rag-1 expression has been reported in intestinal lymphoid cells of common carp [51] and sea bass [49]. This suggests that the possibility of extra-thymic development of T cells in fish intestine.

Recent studies have highlighted the significance of gills as mucosal immune tissues in fish. Haugarvoll *et al.* [52] first demonstrated the presence of intraepithelial cell accumulations on the caudal edge of interbranchial septum at the base of the gill filaments in Atlantic salmon. MHC class II^+^ cells were detected by immunohistochemistry, and TCR mRNA expression was reported by RT-PCR analysis, suggesting the presence of T cells. Koppang *et al.* [6] further confirmed the presence of T cells using sera recognizing a peptide sequence of the CD3ε chain and reported accumulations of T cells in interbranchial lymphoid tissue (ILT). Dalum *et al.* [53] reported higher expression of CD4-1*-* than that of CD8α- related genes in all gill segments investigated and numerous MHC class II^+^ cells throughout the filament epithelial tissue. Interestingly, the higher number of CD4-1^+^ T cells than CD8α^+^ T cells in the gill is in large contrast with the intestine where CD8α^+^ T cells are the dominant population.

In Atlantic salmon examined 17 days post-challenge with ISAV, Hetlandet al. [54] reported the presence of CD8α-positive cells in the gill and a reduction of CD8α and MHC II labelled cells after ISAV infection using antibodies against recombinant proteins from MHC I, II and CD8.

In European sea bass, Ortiz *et al.* [55] reported the presence of considerable numbers of T cells in the gill epithelium where 10%–20% of cells were positive with the T cell-specific mAb DLT15. Leukocytes from gills were able to proliferate in the presence of lectins ConA and PHA and the number of T cells increased during proliferation. In lectin-proliferating cells the expression of T cell-related genes *tcrβ*, *tcrγ*, *cd4*, *cd8α*, *cd45* and *il10* increased dramatically suggesting T cell activity in fish gills.

Recently, Somamoto *et al.* [56] indicated gills may play important roles as vaccination sites for inducing adaptive systemic immunity using a “per-gill infection method”, which directly exposed virus only to gills. The viral load in crucian carp hematopoietic necrosis virus (CHNV)-infected gills decreased after peaking at a particular time point. Gene expression analysis demonstrated that *ifnγ* in gills and *perforin* in kidney increased after the gill infection. CD8^+^ cells among kidney leukocytes increased after the secondary infection, whereas IgM^+^ cells decreased. Collectively, these results suggest that IFN-γ and CTL contribute to controlling CHNV-replication in gills and kidney.

## 5. Function of Fish T Cells

It is well known in mammals that T cells play a central role in adaptive immune response and the several subsets of T cells have a distinct function involved in both humoral and cell-mediated immune responses. In fish similar functions of T cells known for mammals have been reported in *in vivo* and *in vitro* experiments, e.g., Th cells assist other cells such as B cells and macrophages, CTLs kill virus-infected cells and transplanted allogeneic cells and tissues.

### 5.1. In Vivo Studies

#### 5.1.1. Transplantation Studies

Skin and/or scale allograft rejection is a representative phenomenon of *in vivo* specific cell-mediated immunity. Scale grafting technique has been established by Mori [57] to investigate the regeneration of transplanted scales. However, Hildemann [58,59] is a pioneer worker to study the allograft rejection from an immunological point of view. Since then, a number of studies have been reported with regards to rejection process and the time of complete rejection, the effects of temperature, presence of anamnestic response in wide range of species including cyclostomes, elasmobranchs and teleosts [60]. Involvement of T cells in allograft rejection in fish was first demonstrated by Abelli *et al.* [61] in sea bass. Immuno-histochemical studies using mAb DLT15 showed a high density of lymphocytes in allografts and provided evidence for the predominance of T-cells.

Romano *et al.* [62] further characterized the effector cells involved in allograft rejection in sea bass employing electron microscopy combined with FACS, RT-PCR analysis, and ISH. Two different types of T-lymphocytes (DLT15-immunoreactive) infiltrating the allografts were identified and TcRβ^+^ cells in the graft were less numerous compared with DLT15-positive cells. From these results, they suggested that cytotoxic cells might express different TCR phenotypes.

**Figure 1:**
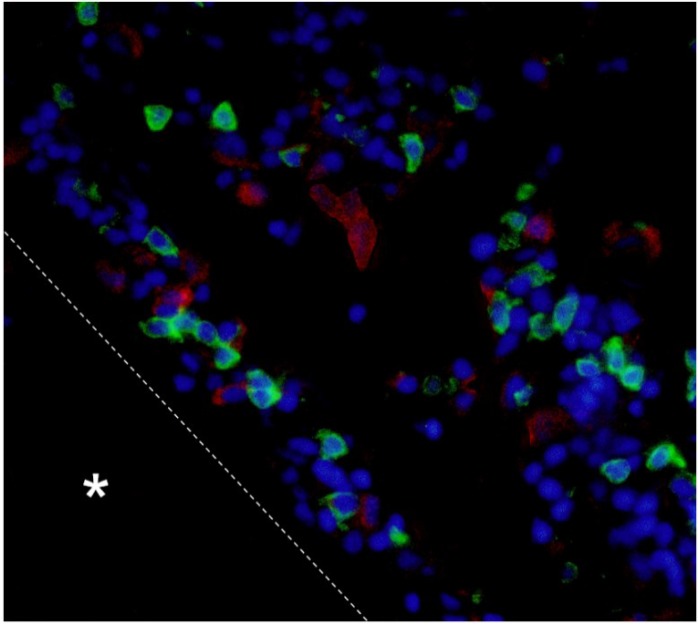
Accumulation of CD4- (red) and CD8α- (green ) positive T cells in 3 days after allografted scales. Nuclei were stained with DAPI (blue). Asterisks and dotted lines indicate grafted scales and their borders respectively. Cited from [63].

Recently, Shibasaki *et al.* [63] reported the kinetics of CD4^+^ and CD8α^+^ T cells along with sIgM^+^ cells and granulocytes/macrophages during allograft rejection using ginbuna crucian carp. They showed that CD4^+^ T cells first infiltrated into allogeneic scales followed by CD8α^+^ and sIgM^+^ cells, and finally phagocytic cells appeared in the graft. Interestingly, most of the CD8α^+^ T cells appeared on the border of the allografted scales at the time of rejection. These results suggest that T cells play crucial roles and work together with other cell types for completion of allograft rejection (Figure 1).

#### 5.1.2. GVHR(GVHD)

The Graft-*Versus*-Host Reaction (GVHR) is a phenomenon of cell-mediated immunity that occurs when tissue grafts contain immunologically competent cells, the recipient cannot recognize or destroy the transplanted cells, and the recipient expresses tissue antigens that are not present in the transplant donor. The presence of GVHR in a teleost fish has been demonstrated employing a model system of clonal triploid ginbuna and tetraploid ginbuna-goldfish (*Carassius auratus*) hybrids [64] and clonal diploid and triploid amago salmon (*Oncorhynchus rhodurus*) [65]. Most features of acute Graft-*Versus*-Host-Disease (GVHD) in ginbuna and amago salmon are quite similar to those reported in mammals, suggesting the existence of similar mechanisms.

Shibasaki *et al.* [66] reported that donor-derived CD8α^+^ T cells play essential roles in the induction of acute GVHR/D in teleosts as in mammals. GVHR was not induced by a leukocyte fraction lacking CD8α^+^ T cells separated by magnetic cell sorting. Ploidy and immunofluorescence analysis revealed that CD4^+^ and CD8α^+^ T cells from sensitized donors greatly increased in the host trunk-kidney, constituting more than 80% of total cells 1–2 weeks after donor cell injection, while those from non-sensitized donors constituted less than 50% of cells present. The increase of CD4^+^ T cells was greater and more rapid than that of CD8α^+^ T cells. Pathologic changes similar to those in human and murine acute GVHD were observed in the lymphoid organs as well as target organs such as skin, liver and intestine, including the destruction of cells and tissues and massive leukocyte infiltration.

### 5.2. In Vitro, Ex Vivo Studies

#### 5.2.1. Helper Function of CD4^+^ T Cells

Conservation of CD4^+^ helper T cell functions among teleost fishes has been suggested in a number studies employing MLC and hapten/carrier effect. MLC is an *in vitro* model to assess the proliferation of Th (responder) cells cultured with MHC class II disparate antigen presenting cells (APCs, stimulator). MLC has been reported in several fish species upon *in vitro* incubation of allogeneic leukocytes [67]. In channel catfish it has been reported that surface Ig-negative (sIg^−^) lymphocytes were the responding cells in MLC [68] and they co-operated with B cells (sIg^+^) and macrophages for *in vitro* antibody responses [69]. In these studies, however, the responding cells were not identified as CD4^+^ T cells, although the involvement of T cells was suggested. Specific proliferation of CD4^+^ T cells after stimulation with alloantigen or thymus-dependent antigen such as ovalubumin (OVA) is also an indicator of Th cell activation. Toda *et al.* [11] demonstrated the proliferation of CD4^+^ T cells and then CD8^+^ T cells following stimulation with allogeneically distinct leukocytes that may have included several cell types of APCs. They also showed antigen-specific proliferation of CD4^+^ T cells after *in vitro* sensitization with the same antigen following pre-sensitization of host fish with OVA.

Somamoto *et al.* [45] have shown that CD4^+^ Th-cells in fish are actually involved in both humoral and cell-mediated immunity during a secondary immune response by adoptive transfer using clonal ginbuna crucian carp and crucian carp hematopoietic necrosis virus (CHNV). Namely, transplanting CHNV-sensitized donor cells, containing CD4^+^ cells, into naive fish induced more rapid and stronger antibody production than those that received non-sensitized donor cells or sensitized donor cells lacking CD4^+^ cells. As for cell-mediated immunity, recipients that received both sensitized donor cell populations (with and without CD4^+^ cells) exhibited more efficient cell-mediated cytotoxicity than those received non-sensitized donor cells. These findings suggest that the secondary antibody response requires CD4^+^ cell help, and secondary cell-mediated immunity can be induced in the presence of either CD4^+^ cells or leukocytes other than CD4^+^ cells.

#### 5.2.2. Cytotoxicity of CD8^+^ T Cells

##### Specific-CMC against Allogeneic Cells and Tissues

**Figure 2:**
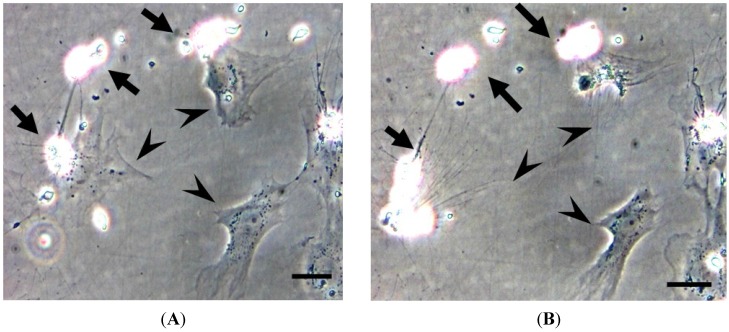
Killing of target cells by allo-sensitized CD8α^+^ cells observed at 3 h (**A**) and 4 h (**B**). The arrow indicates aggregation of effector CD8α^+^ cells. The arrowhead points to target CFS cell. Scale bar = 10 μm. Cited from [72].

TCR αβ^+^ alloantigen-specific cytotoxic cells have been reported in channel catfish but CD8α expression was not examined due to the lack of genetic information on CD8 in that species. Cells involved in alloantigen-specific cytotoxicity have been identified as CD8α^+^ T lymphocytes in ginbuna as mentioned above [10]. This is the first demonstration of the presence of CTLs in a defined T cell subset in fish. Fish CTLs have characteristics similar to those of mammals. For instance, in ginbuna the effector donor must be sensitized by allogeneic tissues and/or by injection of an allogeneic cell line, and cells from non-sensitized fish do not show any significant cytotoxic activity. The cytotoxicity of effector cells correlates well with their expression of CD8α [70,71] (Figure 2).

In channel catfish, cultures initiated with PBL from alloantigen-immunized fish yielded exclusively TCR αβ^+^ cytotoxic cells that included either alloantigen-specific CTLs or cytotoxic cells with broad allogeneic specificity [73], while TCR αβ^−^ non-specific cytotoxic cells were obtained from MLC-generated cultures using PBL from non-immunized fish. Accordingly, pre-sensitization of effector donors is essential to induce CTLs, as in mammals. The requirement for sensitization to detect CMC has been also reported in rainbow trout [74]. CD8α expression was barely detectable in the blood of non-sensitized trout or trout that received xenografts, but was easily detected in the blood of allogeneically stimulated trout. Furthermore, CD8α expression in sIgM^−^ lymphocytes from immunized trout was secondarily enhanced by addition of allogeneic targets *in vitro*.

For the *in vitro* induction of cytotoxic cells, MLC of effector cells with allogeneic stimulator cells is essential in fish as in mammals. Greatly increased (approximately 100-fold) cytotoxic responses were generated by stimulation of channel catfish PBL with irradiated cells of allogeneic cloned B cell lines in MLC. However, MLC-generated cytotoxicity did not exhibit alloantigen specificity, although a considerable number of cytotoxic clones have been established from the MLC [75]. Zhou *et al.* [73] succeeded in obtaining alloantigen specific TCR αβ^+^ cytotoxic clones employing PBL from alloantigen-immunized fish instead of naïve PBL. Similarly, alloantigen-specific cytotoxic cells have been produced in ginbuna. Proliferative responses of responder cells from OB1 strain ginbuna were detected by stimulation with allogeneic cell lines (K1 or S3N stimulator) but not a syngeneic cell line (OB1 stimulator) [76]. The effector cells stimulated with allogeneic cells specifically killed allogeneic targets but not syngeneic targets (CFO-2 cells) (Table 2).

**Table 2:** Identification of CTLs involved in specific cell-mediated cytotoxicity in fish.

Effector cells	Target cells	T-cell markers	Species	References
alloantigen specific cytotoxic T cell clone	Clonal allogeneic B cell line	TCRαβ	Channel catfish	[77]
PBL from immunized fish	IPNV infected syngeneic cell line	Not identified	Ginbuna crucian carp	[78]
alloantigen specific cytotoxic T cell clone	Clonal allogeneic B cell line	TCRαβ	Channel catfish	[73]
Leukocytes from blood or kidney	CHNV infected syngeneic cell line	Not identified	Ginbuna crucian carp	[79]
sIg^−^ lymphocytes from PBL	Allogeneic erythrocytes, RTG-2	TCRα and CD8α	Rainbow trout	[74]
Cytotoxic lymphocytes generated by MLC	Allogeneic cell lines	TCRβ and CD8α	Ginbuna crucian carp	[76]
Leukocytes from anally immunized fish	Allogeneic cell lines (EPC, KG)	Not identified	Carp	[80]
sIg^−^ lymphocytes from kidney	CHNV infected syngeneic cell line	TCRβ and CD8α	Ginbuna crucian carp	[71]
PBL from virus- infected fish	VHSV infected RTG-2	CD8α	Rainbow trout	[81]
PBL from viral DNA immunized trout	VHSV & IHNV infected RTG-2	CD8α	Rainbow trout	[82]
Separated CD8α^+^ T cells by mAb	Allogeneic cell lines	TCRβ and CD8α	Ginbuna crucian carp	[10]
Cytotoxic lymphocytes generated by MLC	CHNV infected syngeneic cell line	TCRβ and CD8α	Ginbuna crucian carp	[83]
PBL from orally immunized fish	CHNV infected syngeneic cell line	TCRβ and CD8α	Ginbuna crucian carp	[84]
Separated CD8α^+^ PBL from NNV sensitized fish by antiserum	NNV infected autologous and allogeneic fin cells	CD8α	Orange-spotted grouper	[85]
Separated CD8α^+^ T cells by mAb	GFP-labeled *E. tarda* phagocytizing leukocytes	CD8α	Ginbuna crucian carp	[86]

##### Specific-CMC against Virus-Infected Cells

CTL-mediated virus-specific cytotoxicity in fish was first described by Somamoto *et al.* [78], although a few earlier papers described the lysis of virus-infected cells by NK-like cells in rainbow trout and channel catfish (See review by [87]). Convincing data showing the essential roles of CTLs against viral infection were reported by Somamoto *et al.* [79]. This is the first demonstration of the primary role of cell-mediated immunity in protecting fish *in vivo* from acute viral infection. Specific CMC of ginbuna leukocytes against hematopoietic necrosis virus (CHNV)-infected syngeneic cells was induced by i.p. injection with CHNV. This cytotoxicity was virus-specific and MHC-restricted, in a manner similar to mammalian CTL activity, since the cytotoxicity was not induced against either virus-infected allogeneic cells or eel rhabdovirus from America (EVA)-infected syngeneic cells. Viral titers in tissues from infected fish were remarkably reduced eight days after infection, when specific cytotoxic activity reached a peak, while CHNV-specific antibody increased only after the virus was eliminated by cytotoxic activities. This result suggested that specific cytotoxic cells rather than antibodies were responsible for the early control of CHNV replication. Furthermore, the effectiveness of the virus-specific cytotoxicity was transferable, since recipients that received leukocytes from immune syngeneic donors escaped CHNV infection.

Somamoto *et al.* [83] further demonstrated an *in vitro* generation of virus-specific cytotoxic T cells in MLC employing ginbuna as a model system. Responder cells (primarily lymphocytes) from CHNV-infected fish were capable of proliferating after stimulation *in vitro* with CHNV-infected syngeneic stimulator cells (primarily lymphocytes and macrophages). The generated effector cells collected eight and 12 days after the *in vitro* stimulation efficiently lysed CHNV-infected syngeneic (MHC class I-matched) cells, but not CHNV-infected allogeneic cells and EVA-infected syngeneic cells. This suggests that the effector cells recognize target in an antigen specific manner as in mammalian CTLs.

More recently, Somamoto *et al.* [88] reported that cytotoxic cells other than CTLs were the dominant effectors, because CTL-depleted peripheral blood leukocytes (PBL) exhibited significant cytotoxic activity against CHNV-infected cells. In addition, the adoptive transfer of CTL-depleted PBL provided as efficient protection against CHNV-infection as the transfer of PBL containing CTLs. Further analyses showed that sIg/CD8α^−^ cells and monocyte-enriched effectors possessed activities that were comparable to or were higher than that of CD8α^+^ cells, suggesting that natural killer (NK)-like cells and monocytes are among the dominant effector cells. CMC inhibition assays with concanamycin A suggested that CTLs and CD8α^−^ lymphocytes lysed virus-infected cells by a perforin-based cytotoxic pathway. These results indicate that CMC induced by viral-infection is executed by not only CTLs but monocytes and CD8α/IgM^−^ lymphocytes.

In rainbow trout Utke *et al.* [81] reported that PBL from low dose viral haemorrhagic septicaemia virus (VHSV)-infected trout killed MHC class I-matched VHSV-infected cells using a system of MHC class I-matched effector and target cells where the allele of classical MHC class I locus Onmy-UBA in rainbow trout clone C25 and in the cell line RTG-2 is identical. However, the PBL also killed xenogeneic MHC class I-mismatched VHSV-infected xenogeneic (EPC) target cells. They also found enhanced mRNA expression of *cd8α* and the natural killer cell enhancement factor (*nkef*)-like gene in the PBL. These results suggest that both NK and cytotoxic T cells are involved in protection against VHSV infection. They further investigated the cell-mediated immune responses in rainbow trout after DNA immunization against the VHSV to identify viral proteins responsible for the induction of the responses. They found that PBL from fish immunized against DNA encoding the VHSV G protein significantly killed VHSV-infected but not IHNV-infected targets, indicating antigen specificity, although the PBL killed both VHSV-infected MHC class I matched (RTG-2) and VHSV-infected xenogeneic target (EPC) cells, suggesting again the involvement of both CTL and NK cells, respectively [82]. The G protein was a more potent trigger of cytotoxic cells than the N protein in VHS DNA vaccine. Interestingly, PBL from trout that were immunized against the N protein killed only VHSV-infected RTG-2 cells, indicating that this protein elicits only an adaptive (CTLs) but not innate (NK cells) immune response.

##### Specific-CMC against Cell-Associated Bacteria

*Edwardsiella tarda* is an intracellular bacterial pathogen that causes edwardsiellosis in fish. Yamasaki *et al.* [86] reported the important role of cell-mediated immunity rather than humoral immunity against intracellular bacterial infection in ginbuna crucian carp. Innate immunity was observed to be the principal immune mechanism for eliminating the majority of *E. tarda*, while a proportion of the bacteria may have been resistant to its bactericidal activity. Bacterial clearance in kidney and spleen was also observed following elevated cytotoxic activity of CTLs and increased numbers of CD8α^+^ cells, suggesting that CTLs might contribute to the elimination of *E. tarda*-infected cells with specific cytotoxicity. In contrast, *E. tarda*-specific antibody titers did not increase until after bacterial clearance, indicating that induction of humoral immunity would be too late to provide protection against infection. Accordingly, these data suggest that both cell-mediated immunity and innate immunity rather than humoral immunity may play important roles in the protection against intracellular bacterial infection, as in mammals.

Yamasaki *et al.* [89] showed the important role of cell-mediated immunity (CMI) in protection against *E. tarda* infection by adoptive transfer of sensitized lymphocytes. They adoptively transferred T-cell subsets sensitized with *E. tarda* to isogenic naïve ginbuna to identify the T-cell subsets involved in protecting fish from infection. Recipients of CD4^+^ and CD8α^+^ cells showed significant resistance to infection with *E. tarda* eight days after sensitization, indicating that helper T cells and cytotoxic T lymphocytes play crucial roles in protection against *E. tarda*. Moreover, transfer of sensitized CD8α^+^ cells up-regulated the expression of *ifnγ* and *perforin* genes, suggesting that protective immunity to *E. tarda* involves cell-mediated cytotoxicity and IFNγ-mediated induction of CMI.

Yamasaki *et al.* [90] further demonstrated the importance of cell-mediated immunity against *E. tarda* infection using vaccine trials comparing the effects of live *versus* formalin-killed bacteria. In their previous studies vaccination with formalin-killed cells (FKC) was not as successful as a live attenuated vaccine in protecting fish against *E. tarda* infection. In order to investigate the mechanism underlying effectiveness they compared the adaptive immune responses in fish vaccinated with FKCs and live attenuated vaccines. After challenge with *E. tarda*, live cell (LC)-vaccinated fish showed high survival rates, high IFNγ and T-bet gene expression levels, and increased CTLs. In contrast, all FKC-vaccinated fish died following *E. tarda* infection. In addition, FKC vaccination induced high il4/13a and *il10* expression levels and increased antibody titers, whereas Th1-like responses were suppressed. These results indicate that LC vaccination contributes to protection against *E. tarda* infection by inducing CMI.

#### 5.2.3. Killing Mechanisms of CTLs

CTLs kill their cellular targets via either of two mechanisms each requiring direct contact between the effector and target cells, *i.e.*, the secretory and non-secretory pathways. Both of these pathways induce apoptosis. The latter pathway involves the engagement and aggregation of target-cell death receptors such as Fas by their cognate ligands, (e.g., Fas ligand, FasL) on the cell surface of effector cells, which results in classical caspase-dependent apoptosis [91]. In the secretory pathway, cytoplasmic granular toxins, predominantly a calcium dependent membrane-disrupting protein known as perforin, and a family of structurally related serine proteases (granzymes) are secreted by exocytosis and together induce apoptosis of the target cell [92,93]. A positive correlation exists between expression of perforin/granzyme and activated mammalian CTLs [94,95].

The FasL protein has been identified in channel catfish, tilapia and gilthead sea bream using anti-human or anti-mouse FasL antibodies [96,97,98]. FasL genes have been isolated in Japanese flounder and zebrafish [99,100]. Recombinant flounder FasL protein induced apoptosis in a Japanese flounder cell line, indicating that fish possess a Fas ligand system [100]. A perforin-like molecule has been identified in Japanese flounder [101], trout [102] and ginbuna [72], and perforin gene expression has been identified in lymphoid tissues. Furthermore, lytic activity of recombinant perforin protein in the presence of calcium has been detected in Japanese flounder [101].

In channel catfish, various types of cytotoxic cells have been reported and killing mechanisms seem to be different between cell types. Killing by group I clones, TCR αβ^+^ alloantigen specific cytotoxic clones that are considered to be catfish equivalent of CTLs, was completely inhibited by treatment with the Ca^2+^-chelating agent EGTA or a perforin inhibitor, concanamycin A (CMA). The killing was sensitive to the serine esterase inhibitor PMSF, while killing by group II clones, TCR αβ^+^ nonspecific cytotoxic clones, was only partially inhibited by EGTA or CMA. These findings suggest that killing by group I cells utilizes the Ca^2+^-dependent perforin/granzyme pathway while group II cells use a pathway involving FasL/Fas or TNF/TNF-R in addition to perforin/granzyme pathway [73]. A major role for the perforin/granzyme pathway in the killing mechanism of alloantigen specific cytotoxic cells has also been reported in lymphocytes from carp immunized with EPC cells [103] and CD8α^+^ lymphocytes from ginbuna immunized with allogeneic scales and cell lines [72], although blocking with EGTA and/or a perforin inhibitor was not complete, indicating the existence of at least one other pathway. In addition, two types of nonspecific cytotoxic cells have been reported in catfish. One is the TCRαβ^−^ nonspecific cytotoxic cell (group IV) that is considered to be a catfish equivalent of NK cells of mice and humans. These cells appear to utilize a perforin/granzyme pathway rather than the Fas/FasL pathway to trigger apoptosis due to the complete inhibition of allogeneic killing by EGTA [104]. The other is the NCC group that lyse sensitive tumor cells by multiple effector pathways including FasL/Fas and perforin/granzyme [96]. Unlike mammalian NK cells and T-cells, activated NCCs do not express membrane FasL. FasL secretion by activated NCCs may function in the presence of FasR positive target cells, since the presence of a soluble form as well as membrane-bound form of FasL has been reported in catfish as in mammals [105]. These studies strongly suggest that pathways of killing similar to those of mammals are operative in fish.

Very recently, a granzyme (Gzms) has been identified and characterized in ginbuna crucian carp, *Carassius auratus langsdorfii* [106]. The primary structure of the granzyme (termed gcGzm) resembled mammalian GzmB, and gcGzm clustered with mammalian GzmB by phylogenetic tree analysis. gcGzm was secreted from HEK293 cells transfected with gcGzm cDNA and was predominantly expressed in CD8^+^ T cells, as in mammals. Expression of gcGzm mRNA was greatly enhanced by allo-sensitization and infection with the intracellular pathogen *Edwardsiella tarda*, indicating that gcGzm is involved in cell-mediated immunity. However, its enzymatic activity was different from mammalian Gzms because gcGzm did not cleave the known substrates for mammalian Gzms. Thus, we conclude that the newly discovered gcGzm is a novel secretory serine protease involved in cell-mediated immunity in fish, with similar structure to human GzmB but different substrate specificity.

#### 5.2.4. Direct Antibacterial Activity of Lymphocytes

The killing mechanism of CTLs against intracellular pathogens involves MHC-restricted and antigen-specific recognition and binding of infected host cells [105,107]. In addition to these activities, recent studies in mammals have suggested that CTLs can exhibit direct antimicrobial activity and can kill different types of pathogens including bacteria, parasites, and fungi [108]. In contrast to killing of tumor and microbe-infected cells, direct killing of extracellular pathogens by CTLs is apparently an MHC-independent event since microorganisms do not express MHC. Recognition and killing mechanisms in direct microbicidal activity of CTLs are largely unknown even in mammals, although specific recognition of antigen via MHC and the killing mechanisms of infected host cells by CTLs are well-known.

Nayak *et al.* [109] demonstrated the direct antibacterial activity of CD8α^+^, CD4^+^ T-cells and sIgM^+^ cells in fish. The CD8α^+^ T cells from sensitized ginbuna exhibited antibacterial activity against both cell-associated and extracellular bacteria. The maximum reduction of viable count of pathogens was recorded with effector (sensitized) cells and target (bacteria) ratio of 10:1 co-incubated for a period of 1–2 h at 26 °C when effector cells were derived from ginbuna 7 days after a booster dose given on the 15th day following primary sensitization/immunization. Sensitized CD8α^+^ T cells were found to kill bacteria used for immunogen, e.g., 92.1 and 98.9% of *Lactococcus garvie* and *Edwardiella tarda*, respectively. No significant difference in bacterial killing activity could be detected against cell-associated and extracellular bacteria. However, CD8α^+^ T cells from *E. tarda* immunized ginbuna exhibited 40% of the non-specific killing against *L. garvie* and those from *L. garvie* immunized ginbuna showed 42.7% of the non-specific killing against *E. tarda*. Furthermore, CD4^+^ T cells also killed 88% and 95.7% of *L. garvie* and *E. tarda*, respectively. In addition to T cell subsets, surface Ig M^+^ cells (presumably NK cells with Fc receptor) also killed both types of pathogens.

In our study above, we documented direct antibacterial activity of lymphocytes from immunized fish. However, we also discovered weak non-specific killing activity of lymphocytes against bacteria. We further analyzed the weak killing activity of lymphocytes, increasing the effector cell to target bacteria ratio from 10:1 to 10^3^:1 [110]. Sensitized and non-sensitized effector lymphocytes (CD8α^+^, CD4^+^ and sIgM^+^) separated by MACS were incubated with target bacteria. CD8α^+^ T-cells from *E. tarda*-immunized ginbuna crucian carp killed 98%, 100% and 70% of *E. tarda*, *Streptococcus iniae* and *Escherichia coli*, respectively. CD8α^+^ T-cells from non-immunized fish showed similar but slightly lower killing activity than sensitized cells. CD4^+^ and sIgM^+^ lymphocytes also showed high killing activity against *E. tarda* and *S. iniae* as found for CD8α^+^ T-cells, although the activity was lower against *E. coli*. Supernatants from all three types of lymphocytes showed microbicidal activity, although the activity was lower than that evoked by effector lymphocytes. Furthermore, the presence of a membrane between effectors and targets did not affect the killing activity. These results suggest that both sensitized and non-sensitized lymphocytes non-specifically killed target bacteria without the need for contact. The major difference between the present and previous experiments is the E:T ratio. We suspect that there are two different mechanisms in the direct bacterial killing by lymphocytes in ginbuna.

## 6. Future Directions

As mentioned in our previous review [111], new immunological reagents and cytotoxic and/or helper T cell clones are essential for further development in this field. We recently found that our mAbs against CD4-1 and CD8α in ginbuna crucian carp cross react with zebrafish lymphocytes. First, both 6D1 (CD4-1) and 2C3 (CD8α) recognized approximately 10% of zebrafish lymphocytes that were all ZAP70^+^ by dual fluorescence analysis. Second, FACS sorted 6D1^+^ lymphocytes express *cd4-1* and *tcrα* but not *cd8α* and *igl*, while 2C3^+^ lymphocytes express *cd8α* and *tcrα* but not *cd4-1* and *igl*. Furthermore, 6D1 (CD4-1) and 2C3 (CD8α) mAbs react with zebrafish CD4-1 and CD8α expressed on HEK293T cells, respectively. These findings suggest that mAbs against ginbuna CD4-1 and CD8α can be used to identify T cell subsets of zebrafish as well as the cyprinids goldfish and carp. The zebrafish is emerging as a model species not only for fish but also for humans. Therefore, these mAbs will surely be useful for the identification and characterization of zebrafish T cell subsets.

It has been reported that there exist fish-specific CD4 (CD4rel or CD4-2) in addition to CD4 (CD4-1) homologous to mammalian CD4 [112,113]. Likewise, fish-specific IFNγ (IFNγ rel) is present in several fish species [114,115]. T helper cell differentiation, particularly in the balance between Th1 and Th2 cells, has not been demonstrated in fish, although mAbs against CD4 to isolate helper T cells are available in several fish species, e.g., ginbuna and rainbow trout, along with recombinant IL-12 (p35, p40) and antibodies against IFNγ. It would be quite interesting to know which isoforms of CD4 and/or IFNγ are involved in the differentiation of naïve T cells toward a Th1 fate.

As mentioned in the section “Specific-CMC against virus-infected cells”, Somamoto *et al.* [88] reported that cytotoxic cells other than CTLs were the dominant effectors against viral-infected cells. They suggested that natural killer (NK)-like cells and monocytes are among the dominant effector cells. However, identification and characterization of NK cells in fish other than channel catfish has been hampered by a lack of suitable cell surface markers. Involvement of NK cells in the killing activities against allogeneic cells [10] and in direct killing [109] as well as viral-infected cells has been suggested. There is also the possibility of the presence of intermediate cell types between NK cells and T cells in fish, like NKT cells in mammals. Therefore, identification and separation of NK cells are essential to understand the major cell types involved in cell-mediated immunity.
